# Phylogeography of *Thlaspi arvense* (Brassicaceae) in China Inferred from Chloroplast and Nuclear DNA Sequences and Ecological Niche Modeling

**DOI:** 10.3390/ijms160613339

**Published:** 2015-06-11

**Authors:** Miao An, Liyan Zeng, Ticao Zhang, Yang Zhong

**Affiliations:** 1 School of Life Sciences, Fudan University, Shanghai 200433, China; E-Mail: 11110700011@fudan.edu.cn; 2 Shanghai Chenshan Plant Science Research Center, Chinese Academy of Sciences/Shanghai Chenshan Botanical Garden, Shanghai 201602, China; 3 Shanghai Public Health Clinical Center, Fudan University, Shanghai 201508, China; E-Mail: ziqi1103@163.com; 4 Key Laboratory for Plant Diversity and Biogeography of East Asia, Kunming Institute of Botany, CAS, Kunming 650201, China; 5 Institute of Biodiversity Science and Geobiology, Tibet University, Lhasa 850000, China

**Keywords:** Qinghai-Tibetan Plateau, population structure, admixture, molecular dating, MaxEnt

## Abstract

*Thlaspi arvense* is a well-known annual farmland weed with worldwide distribution, which can be found from sea level to above 4000 m high on the Qinghai-Tibetan Plateau (QTP). In this paper, a phylogeographic history of *T. arvense* including 19 populations from China was inferred by using three chloroplast (cp) DNA segments (*trnL-trnF*, *rpl32-trnL* and *rps16*) and one nuclear (*n*) DNA segment (Fe-regulated transporter-like protein, *ZIP*). A total of 11 chloroplast haplotypes and six nuclear alleles were identified, and haplotypes unique to the QTP were recognized (C4, C5, C7 and N4). On the basis of molecular dating, haplotypes C4, C5 and C7 have separated from others around 1.58 Ma for cpDNA, which corresponds to the QTP uplift. In addition, this article suggests that the *T. arvense* populations in China are a mixture of diverged subpopulations as inferred by hT/vT test (hT ≤ vT, cpDNA) and positive Tajima’s D values (1.87, 0.05 < *p* < 0.10 for cpDNA and 3.37, *p* < 0.01 for nDNA). Multimodality mismatch distribution curves and a relatively large shared area of suitable environmental conditions between the Last Glacial Maximum (LGM) as well as the present time recognized by MaxEnt software reject the sudden expansion population model.

## 1. Introduction

The uprising of the Qinghai-Tibetan Plateau (QTP) is considered to have resulted in extensive genetic divergence [1], because it is the largest, highest, one of the youngest, and one of the most extensive plateaus on earth [1,2]. The QTP has continued to rise from the Late Tertiary/mid-Miocene to the Quaternary [3,4,5]. During the uplift period, the most drastic episode is within about 1.2–0.6 million years ago (Ma) and is called the “Kunhuang movement” [3,6]. The violent uplift initiated drastic climate shifts and formation of glaciers, consequently limiting the spread of many species and hence their range was reduced and partitioned. The habitat fragmentation has accelerated both interspecific e.g., [7,8,9] and intraspecific differentiation e.g., [2,10,11].

*Thlaspi arvense*, one of the Brassicaceae species, can adapt to various environmental conditions from sea level to about 4000 m above sea level. *Thlaspi arvense* is native to Eurasia and widely introduced into temperate regions of the northern hemisphere [12]. The genome of *T. arvense* (2*n* = 14) is approximately 539 Mbp [13]. A transcriptome with 33,873 contigs is now available on the NCBI TSA (Transcriptome Shotgun Assembly) database (Genbank: GAKE00000000.1) [14]. As a prolific seed producer, *T. arvense* usually has a short life cycle of two or three months. It is self-compatible and basically self-pollinated, but outcrossing can occur at a rate of 10%–20% [12]. *Thlaspi arvense* has been widely studied, in the context of topics such as enrichment of heavy metals [15,16], regulating effect of gibberellins [17,18], and biological resources [19]. Moreover, the adaptive traits of *T. arvense* have been discussed in a few studies, including cold response [20,21] and flowering time variation [22,23]. However, these traits do not focus on the population level, calling for a phylogeographic investigation of *T. arvense* to provide a research background.

In this study, the transcriptome of *T. arvense* was used to develop nuclear sequence markers. Huang *et al.* has listed frequently-used nuclear markers and has estimated their evolutionary rates [24]. Fe-regulated transporter-like protein (*ZIP* gene) has a relatively fast evolutionary rate. However, *ZIP* is not a single copy gene. Fortunately, although the sequences in the coding region have a great similarity between gene copies, the UTR regions are usually unique [25]. Using a BLAST search against the transcriptome, we found that *ZIP* has two copies in *T. arvense*. In order to apply *ZIP* to the phylogeographic study, when designing primers, at least one primer was designed in the UTR region to ensure that the PCR products are generally homogeneous. Additionally, we use Ecological Niche Modeling (ENM) to supplement the results of the molecular approaches. By reconstructing potential geographic distribution of species during different historical periods, ENM can provide innovative insights in questions in ecology and evolution [26,27,28]. By exploring phylogeographic structure and paleoclimatic influence of *T. arvense*, we addressed three questions: (1) what is the diversity and genetic structure of *T. arvense*; (2) did the uplift of the QTP have an impact on the phylogeographic pattern of *T. arvense*; and (3) how did *T. arvense* respond to the climate fluctuations during the last glacial period.

## 2. Results

### 2.1. Sequence Variation of T. arvense cpDNA and ZIP

Three cpDNA segments from each of 224 *T. arvense* individuals were sequenced. The length of aligned sequences of *trnL-trnF*, *rpl32-trnL*, and *rps16* were 672, 702, and 716 bp, identifying six, three and four chloroplast haplotypes respectively (KJ480797–KJ480809). The *rpl32-trnL* sequences contain five-base inverted-repeat mutations, which were treated as a single mutation. The combined cpDNA sequence was 2086 bp in length with nine nucleotide substitutions, detecting a total of 11 chloroplast haplotypes (C1–C11).

For the *ZIP* gene, 210 individuals were sequenced. The sequence of 1962 bp in length includes two partial exons and an intron. There are ten polymorphic sites in the *ZIP* gene which defined six nuclear alleles N1–N6 (KJ480810–KJ480815). The nucleotide diversity (π) and haplotype diversity (Hd) for each population were estimated (Table 1).

**Figure 1:**
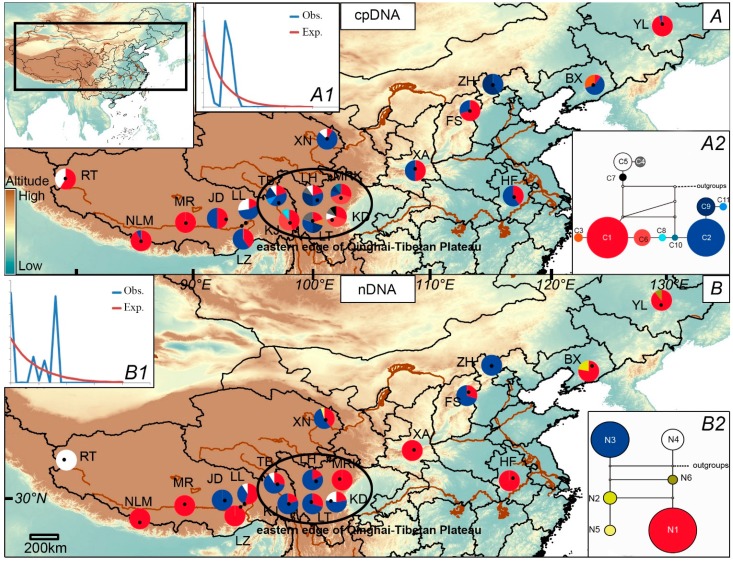
Haplotype distribution of cpDNA (**A**) and *ZIP* (**B**) in *Thlaspi arvense*. Pie charts show haplotype proportions in each population. Multimodality mismatch distribution curves of cpDNA and *ZIP* in the overall populations are shown in A1 and B1. The networks of the 11 chloroplast haplotypes and six nuclear alleles are shown in A2 and B2, separately. Black dots represent missing haplotypes.

The geographical distribution of chloroplast haplotypes and the *ZIP* alleles is illustrated in Figure 1A,B. For cpDNA, the most common haplotypes were C1 and C2. Almost all populations (14 of 19) contained C1 and C2 at the same time. Similarly, for the *ZIP* gene, N1 and N3 were the most common alleles. Eight of 19 populations contained both N1 and N3. Chloroplast haplotypes C4, C5, C7 and nuclear alleles N4 can only be found in the QTP. The details of haplotype distribution for each population are summarized in Table 1.

### 2.2. Population Demography and Phylogeographic Structure

Parameters including N_ST_, G_ST_, vT and hT of both the whole populations and populations in the eastern edge of the QTP are presented in Table 2. Both markers from cpDNA and *ZIP* showed that N_ST_ are slightly higher than G_ST_, but not significantly (*p* > 0.05), showing no strong phylogeographic pattern can be found [29]. The observed multimodal mismatch distributions of the overall populations for both of the two datasets (Figure 1A1,B1) indicated a non-expansion hypothesis. The significant sum of squared deviations (SSD) value (0.10, *p* = 0.01 for cpDNA and 0.21, *p* = 0 for nDNA) and the raggedness index (0.17, *p* = 0.03 for cpDNA and 0.49, *p* = 0), along with positive values of Tajima’s D (1.87, 0.05 < *p* < 0.10 for cpDNA, 3.37, *p* < 0.01 for nDNA) reject a sudden expansion model. Positive Tajima’s D could cause by population admixture. This speculation is supported by hT and vT test. For cpDNA data, vT (0.721) is slightly higher than hT (0.719), but for the *ZIP* data vT equals to hT (Table 2).

**Table 1:** Sampling information, haplotypes and frequencies, nucleotide diversity (π) and haplotype diversity (Hd) of 19 *Thlaspi arvense* populations.

Code	Locality (All in China)	Long.	Lat.	Alt.	cpDNA			nDNA		
		(E)	(N)	(m)	Chloroplast Haplotypes (No.)	π	Hd	Nuclear Alleles (No.)	π	Hd
LZ	Bujiu, Tibet	94°24ʹ	29°28ʹ	2985	C1(2), C2(3)	0.00115	0.600	N1(5)	0.00000	0.000
MRK	Maerkang, Sichuan	102°42ʹ	31°46ʹ	3180	C1(3), C2(1), C6(4), C9(1), C10(1)	0.00106	0.800	N1(2), N3(8)	0.00145	0.356
LH	Luhuo, Sichuan	100°43ʹ	31°36ʹ	3447	C1(2), C2(3), C4(1), C5(1), C9(3)	0.00162	0.844	N1(2), N3(9)	0.00133	0.327
KJ *	Kajun, Sichuan	98°27ʹ	29°43ʹ	3806	C1(4), C6(4), C8(1)	0.00037	0.667	N1(1), N3(4)	0.00091	0.222
TB	Tuoba, Sichuan	97°29ʹ	31°22ʹ	3751	C1(2), C2(4), C5(1), C9(2), C11(1)	0.00133	0.822	N1(3), N3(7), N4(1)	0.00198	0.564
XN	Xining,Qinghai	101°45ʹ	36°38ʹ	2245	C1(2), C2(18), C5(3)	0.00084	0.379	N1(8), N3(11), N5(1)	0.00214	0.563
LT	Litang, Sichuan	100°19ʹ	29°52ʹ	4045	C1(2), C2(3), C4(2), C6(1), C9(2)	0.00176	0.867	N1(3), N3(7)	0.00190	0.467
KD	Kangding, Sichuan	101°57ʹ	29°57ʹ	3180	C1(3), C4(1), C5(1), C6(4), C7(1)	0.00133	0.800	N1(2), N3(5), N4(2)	0.00204	0.667
LL *	Lulang, Tibet	94°43ʹ	29°43ʹ	3436	C1(2), C2(6), C5(3)	0.00147	0.655	N1(6), N3(4), N4(1)	0.00226	0.618
NLM	Nyalam, Tibet	85°58ʹ	28°9ʹ	3763	C1(12), C2(1)	0.00030	0.154	N1(8)	0.00000	0.000
MR *	MangRe, Tibet	89°44ʹ	29°42ʹ	4406	C1(10)	0.00000	0.000	N1(10)	0.00000	0.000
JD *	Jiangda, Tibet	93°4ʹ	29°59ʹ	3570	C1(3), C2(3)	0.00115	0.600	N3(6)	0.00000	0.000
RT *	Ritu, Tibet	79°38ʹ	33°25ʹ	4292	C1(10), C5(7)	0.00123	0.515	N4(13)	0.00000	0.000
HF	Hefei, Anhui	117°11ʹ	31°52ʹ	31	C1(8), C2(12)	0.00097	0.505	N1(13)	0.00000	0.000
ZH	Zhanhai, Hebei	115°24ʹ	41°14ʹ	1771	C2(4), C9(6)	0.00026	0.533	N3(10)	0.00000	0.000
XA *	Xiʹan, Shannxi	109°7ʹ	34°1ʹ	766	C1(5), C2(5)	0.00107	0.556	N1(7)	0.00000	0.000
YL *	Yilan, Harbin	129°34ʹ	46°19ʹ	101	C1(19), C2(1)	0.00019	0.100	N1(17), N2(2)	0.00041	0.199
BX	Benxi, Liaoning	123°47ʹ	41°15ʹ	284	C1(1), C2(6), C3(3)	0.00125	0.600	N1(7), N2(2)	0.00079	0.389
FS	Fansi, Shanxi	113°29ʹ	39°3ʹ	2063	C1(7), C2(3)	0.00090	0.467	N1(3), N3(7)	0.00190	0.467
Total					C1~C11(224)	0.00132	0.696	N1~N6(210)	0.00211	0.604

Samples which were collected in the farmland are marked with asterisk (*).

**Table 2:** Parameters of population diversity in all populations and those positioned at the eastern edge of the Qinghai-Tibetan Plateau (QTP).

	N_ST_	G_ST_	hT	vT	π	Hd
**cpDNA**						
All populations	0.285	0.235	0.719	0.721	0.00132	0.696
Eastern edge of the QTP	0.197	0.041	0.834	0.855	0.00151	0.831
***ZIP***						
All populations	0.565	0.560	0.600	0.600	0.00211	0.604
Eastern edge of the QTP	N/A	N/A	N/A	N/A	0.00154	0.420

N/A: not applicable.

### 2.3. Phylogenetic Analyses and Divergence Time

**Figure 2:**
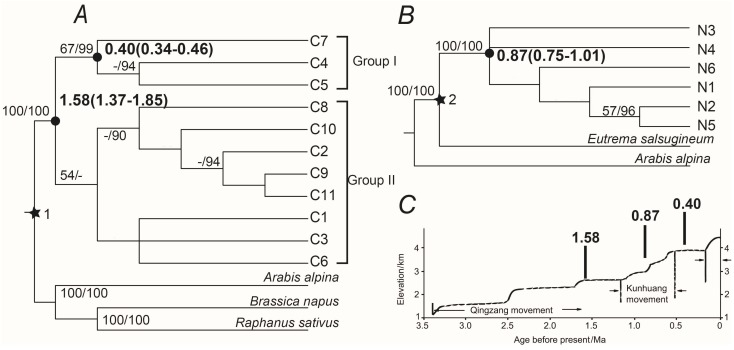
Phylogenetic trees produced by Bayesian inference of cpDNA and *ZIP* of *Thlaspi arvense* are presented in (**A**) and (**B**) Numbers above the branches indicate the bootstrap values for ML/BI analyses. Dash lines represent bootstrap values that lower than 50 or posterior probabilities that lower than 90. The divergence times between *T. arvense* and outgroups referenced the work of Beilstein *et al.* [30] and were marked as stars. Star 1 indicates 38.4 Ma (33.2–45.0 Ma) and star 2 indicates 35.9 Ma (31.1–41.7 Ma). Inferred dates in Ma before present are given beside the nodes denoted with black dots. Stepwise uplift of the Qinghai-Tibetan Plateau (**C**) is adapted from Shi *et al.* [5].

For cpDNA, both Maximum Likelyhood (ML) and Bayesian Inference (BI) trees indicated that two divergent lineages exist in *T. arvense* populations in China (Figure 2A). One lineage contains chloroplast haplotypes C4, C5 and C7 (Group I), which can only be found in the QTP, while another lineage contains the other haplotypes (Group II). Group I obtained a poor bootstrap support value (67%) in ML analysis and a strong posterior probability (99%) in BI analysis. But Group II only obtained very poor support by bootstrap (54%) in ML analysis and poor posterior probability of 76% in BI analysis. For the *ZIP* gene, all nuclear alleles constituted almost a polytomy in ML analysis with very low bootstrap supports or posterior probabilities (Figure 2B).

The BASEML program of PAML version 4 was used to estimate divergent times between *T. arvense* haplotypes. Only the nodes that acquire high bootstrap values or posterior probabilities were labeled with divergence times (Figure 2A,B). For cpDNA, the time to the most recent common ancestor (TMRCA) was dating at 1.58 (1.37–1.85) Ma, and haplotype C4, C5 and C7 differentiated at 0.40 (0.34–0.46) Ma. For the *ZIP* gene, TMRCA was dated at 0.87 (0.75–1.01) Ma. These dates were also marked above the time-line of the QTP uplift (Figure 2C) adapted from Shi *et al.* [5].

For cpDNA, the network showed two divergent lineages, which can correspond to Group I and Group II identified by ML and BI analyses. C1 and C2, the most common haplotypes, were linked by rare haplotypes including C6, C8 and C10. Outgroups were linked at one of five missing haplotypes (Figure 1A2). For the ZIP gene, N1, N3, N4 and N6 were all linked to missing haplotypes. N4 and N6 had most close relationships to outgroups (Figure 1B2).

### 2.4. Past and Present Distributions

**Figure 3:**
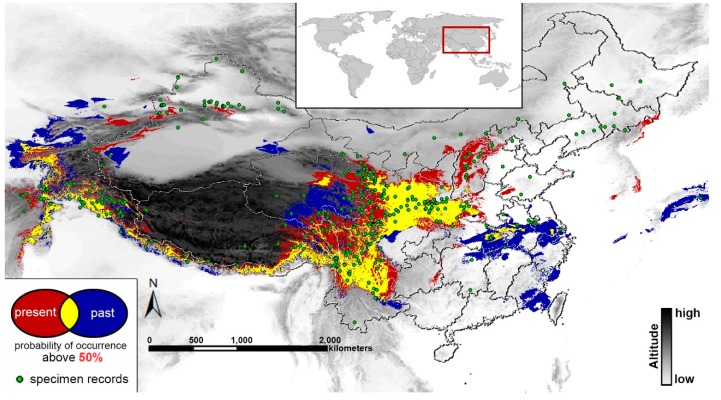
The present (**red**) and past distribution (**blue**) of *T. arvense* predicted through ecological niche modeling by the software MaxEnt3.3.3k. Only area with a predicted suitability above 50% is shown. Yellow represents the shared area of suitable environmental conditions of both the present and the past. Green dots represent the sampling records used for MaxEnt.

The present and past range of *T. arvense* was predicted through the bioclimatic niche modeling showed in Figure 3. Although the noticeable range shift (separate red or blue) occurred at a lower elevation in the eastern China, there is no considerable change between the present and the LGM. To visually view the range changes between the present and the past, the predict distributions (above 50%) were synthesized to produce an integrated result which shown on Figure 3. Yellow represents the shared area of suitable environmental conditions of both the present and the past, while red stands for the area that only exists in the present and blue stands for the area that existed only in the past. The AUC (area under the curve) score for the climate modeling was high at 0.941, which presented a good simulation.

## 3. Discussion

### 3.1. Haplotype Divergence in T. arvense

Group I and N4 only existed in the region of the QTP (Figure 1). For cpDNA, although a majority of haplotypes have weak bootstrap supports in phylogenetic relationships, Group I separated from other haplotypes at about 1.58 Ma with strong bootstrap support (Figure 2A). This date is consistent with the study of *Hippophae tibetana* [2]. In the study [2], haplotypes in the west of the QTP (clade B) separated from others at 1.52 Ma. It was after the third phase of the QTP uplift (about 1.70 Ma) and the plateau reached to 2500 m in an average height. The drastic climate change brought by plateau uplift may cause a natural process of habitat fragmentation, and may produce barriers of gene flow which is the main reason for the genetic differentiation among haplotypes. For the *ZIP* gene, the relationships between six alleles were not solved well by the ML and BI method (Figure 2B), but the network suggested that N4 has a close relationship with the outgroup (Figure 1B2), showing that the divergence of N4 was earlier than other alleles. Although similar phylogeographic structures were presented by cpDNA and *ZIP*, TMRCA of cpDNA (1.58 Ma) is earlier than *ZIP* (0.87 Ma) (Figure 2). This is because cpDNA and nuclear markers differ in modes of inheritance, for example, biparental *vs.* maternal inheritance, effective size and recombination. The two types of markers can reveal different population history. According to previous studies, the time points (0.87 Ma) identified by the *ZIP* gene located at the episode during the QTP uplift named Kunhuang movement [31]. It occurred between 1.2–0.6 Ma and uplifted the plateau to 3000 m in an average height, which is a critical height for glacial development. The plateau has therefore undergone glaciations since the Kunhuang movement as well as climate change [31,32]. Besides, haplotype C4, C5 and C7 differentiated at 0.40 Ma (Figure 2A). After the Kunhuang movement, the QTP experienced a period of relative stability (Figure 2C). It may have created the conditions for population expansion and the following genetic differentiation. 

In general, our study suggested that C4, C5, C7 or N4 are the QTP specific haplotypes, and haplotypes C4, C5 and C7 may has differentiated from others triggered by the QTP uplift. The discovery of the QTP specific haplotypes may provide good material for the study of high-altitude adaptation. However, the dating results may not be accurate because the outgroup species are too divergent with respect to *T. arvense*. Often, outgroup species should preferably be the sister group of the ingroup, but the other *Thlaspi* species are rare in China and difficult to collect. The improper selection of outgroup can result in “random outgroup effect” and long branch attraction [33,34], which would lead to inaccurate results.

Two chloroplast haplotype C1 and C2 were identified from cpDNA. Similarly, two *ZIP* alleles with high frequency (N1 and N3) are identified. *Thlaspi arvense* is globally distributed, but we only sampled the populations circulating in China. Therefore, the origins of two chloroplast haplotypes or nuclear alleles could not be inferred. According to field investigations, *T. arvense* is likely native to Eurasia and then widely spread over the northern hemisphere such as the north America and Canada [12].

### 3.2. Admixture Region of Diverged Haplotypes

According to the phylogeny tree (Figure 2A) of cpDNA, 11 chloroplast haplotypes can be grouped into two clades (Group I and II) with high bootstrap supports. However, we failed to detect any phylogeographic structure neither in the map of haplotype distribution nor in N_ST_/G_ST_ test (*p* > 0.05) [29]. The value of vT (0.721) shows slightly higher than hT (0.719) for cpDNA but equal for *ZIP* (0.600 for both), suggesting that subpopulation admixture may exists [35,36]. Besides, Tajima’s D of both cpDNA and *ZIP* shows positive values (1.87, *p* > 0.05 for cpDNA, 3.37, *p* < 0.05 for *ZIP*). Significant positive value of Tajima’s D means excess of intermediate frequency variants which may be cause by population admixture [37,38,39]. As a result, it is possible that the *T. arvense* population in China is a mixture by highly diverged ancestral subpopulations.

In many other studies around the QTP and mainland China, the differentiated haplotypes are usually grouped well by geographical distances *i.e.*, [7,11]. But no phylogeographic structure was detected in this study. We suspect that it may due to the seed spread at a fast speed. The results show a relatively low differentiation between *T. arvense* populations (G_ST_ = 0.285, cpDNA) when comparing to other sympatric species reviewed in [1]. Most Brassicaceae species are prolific seeders and can spread a long distance, that may have relatively low genetic differentiation, such as *Arabis alpine* [40] and *Arabidopsis thaliana* [41,42]. *Thlaspi arvense* can produce 1600 to 15,000 seeds per plant on average, which can float in water for 24 h [43]. The seeds also have been found to be carried by birds [44]. Except the natural mechanism of dispersal, human activity is considered to be another important factor in seed dispersal [12]. In brief, it is possible that relatively low genetic differentiation and week genetic structure may be the result of frequent seed exchange, that is, gene flow.

Secondary contact of subpopulations can cause increased genetic diversity [45]. This scenario can act to confound with refugia area when speculating refugia from a phylogeographic study. One major feature that differentiates them is that refugia tend to contain private haplotypes. These haplotypes may not participate in the recolonization process and thus cannot be found elsewhere. Furthermore, haplotypes in refugia often have a relatively close genealogical relationship, while in admixture region haplotypes are genetically diverged [45]. In this study, populations in the eastern edge of the QTP have the highest allelic richness (Table 2). These populations are located within the range of Hengduan Mountains which has long been considered as the center of biodiversity and glaciers refugia in China [46,47,48]. However, the result of this study suggests that *T. arvense* populations in the eastern edge of the QTP are more like an admixture region of differentiated haplotypes than glacial refugia due to higher vT values. The value of vT (0.855) is slightly higher than hT (0.834) for cpDNA in the eastern edge of the QTP (Table 2). Furthermore, when seen from the composition of chloroplast haplotypes or the *ZIP* alleles, populations in the eastern edge of the QTP contain all three separated cpDNA lineages or all the high-frequency *ZIP* alleles. The three lineages or alleles may not have a close genealogical relationship. Therefore, populations in the eastern edge of the QTP are more in accordance with the description of admixture. In conclusion, the region of the eastern edge of the QTP is considered to be an admixture zone rather than a glacial refugium.

### 3.3. Ecological Niche Modeling

Ecological Niche Modeling has been widely used to deduce potential distributions for species [26]. By comparison of the simulation results of the LGM and present (Figure 3), the areas of the present have almost the same size as the LGM. The main concentration of the shared areas of suitable environmental conditions (yellow) appeared in the southern (the south of the Himalayas) and eastern edge of the QTP (the Hengduan Mountains), as well as the mountainous region in central China (the Qinling Mountains, the Dabie Mountains, *etc.*). Harrison, *et al*. [49] has used the data of sub-recent pollen and present climate to estimate the potential prehistoric vegetation. They suggested that the above regions were dominated by warm-temperate evergreen forest or temperate deciduous forest during the LGM, and thus they are likely to be the proper habitat for *T. arvense* for the LGM period. Moreover, previous phylogeographic studies of plant species found that their LGM refugia were mainly located in the eastern or southeastern edge of the QTP (e.g., the Hengduan Mountains) [46,47,48], as well as in the southern slope of the Himalaya [11,50,51]. Therefore, the ENM modeling is convincing.

The shared region (marked as yellow) occupies a large proportion of simulation area (Figure 3). It can be interpreted that the *T. arvense* population is less likely to be seriously affected by climatic fluctuations. It is generally accepted that the climate fluctuations between the glacial and interglacial period will promote range shifts at a large scale in an expansion-contraction pattern to plant species [52]. However, an extensive and unified ice-sheet has never directly impacted mainland China [53], and temperate deciduous forest covered the south of China during the LGM according to the pollen data [49]. Thus, the impact of glacial oscillations may be limited to *T. arvense* which has a relatively strong adaptability to environmental changes. However, obvious northward range shifts occurred in the low altitude regions inferred by MaxEnt modeling. The molecular data failed to detect the population expansion in the north China due to the limited sampling.

## 4. Experimental Section

### 4.1. Population Sampling

Leaf samples of a total of 224 individuals were collected from 19 *T. arvense* natural populations in China (Table 1). In each population, individuals were spaced at least 10 m apart from each other. GPS records and voucher specimens were also collected. Leave samples were dried and stored into silica gel immediately after field sampling. To avoid interference from human activity as far as possible, natural distribution was set to the prioritization. Samples collected in the farmland are marked with asterisks (Table 1).

### 4.2. Identification of Nuclear Marker

We chose *ZIP* as the nuclear marker for phylogeography study because it has a relatively fast evolutionary rate in Brassicaceae [24]. We used the sequence of the *ZIP* gene of *Arabidopsis thaliana* (Genbank ID: NM_119456.1) as query to execute BLASTN program of BLAST+ [54] against the TSA database of *T. arvense* [14]. Two homologous *ZIP* genes were obtained which GenBank ID are GAKE01013726 and GAKE01002282, and the latter was selected as nuclear marker in this article. PCR and sequencing primers were designed within 5ʹUTR and 3ʹUTR region (*ZIP*F: TCTTGGGTTTACGAGGATT and *ZIP*R: GCTATAAAAGAACCAATGGAA) to avoid the amplification from the other homologous *ZIP* gene. An additional inner primer was designed in order to complete the sequencing (*ZIP*M: CCGACGGTAGCCTCTTTGTGG).

### 4.3. DNA Extraction, Amplification and Sequencing

Total genomic DNA was extracted from silica-dried leaf tissue by using plant genomic DNA extraction kit (TIANGEN, Beijing, China) following by the protocol. Three non-coding chloroplast DNA (cpDNA) regions: *trnL-trnF* [55], *trnL-rpl32f* [56], *rps16* [57] and one nuclear DNA (nDNA) segment *ZIP* were amplified by polymerase chain reaction (PCR). The PCR amplifications for cpDNA and the *ZIP* genes used the following procedure: 5 min at 94 °C, 35 cycles of 40 s at 94 °C, 30 s at 55 °C, 1 min (for cpDNA) and 5 min (for the *ZIP* gene) elongation at 72 °C, ending with 7 min extension at 72 °C. PCR reactions were carried out in 50 μL containing 25 μL TIANGEN PCR Master Mix (TIANGEN, Beijing, China), 0.3 μL/L each primer and 30–50 ng genomic DNA. The products were purified and sequenced by a commercial laboratory (Majorbio, Shanghai, China). Sequencing chromatograms were checked using Sequencher version 4.1.4 (Gene Codes Corporation, Ann Arbor, MI, USA), then the sequences were aligned using CLUSTALW [58]. All three cpDNA sequences were combined by using a Perl script.

### 4.4. Phylogenetic Analyses

Chloroplast haplotypes and nuclear alleles were assigned by using DnaSP version 5.0 [59]. As the *ZIP* gene is diploid, only four individuals have dinucleotide ambiguities. PHASE program as supplement in DnaSP version 5.0 [59] was used in order to reconstruct the phases of the *ZIP* gene. Phylogenetic analyses of chloroplast haplotype and the *ZIP* alleles were carried out by two methods: ML and BI. ML analyses were conducted using RAXML 7.2.8 [60] under the GTRGAMMAI substitution model. A 1000 “fast bootstrap” replicates were used to assess node support replicates. BI analyses were conducted using MrBayes v.3.1.2 [61]. Runs for cpDNA and *ZIP* began with a random starting tree, and ran for 20,000,000 generations with sampling in each 1000 generations. An initial 25% of the sampled trees were discarded (burnin = 5000). The cpDNA sequences (*trnL-trnF*, *trnL-rpl32*, and *rps16*) of three outgroups (*Brassica napus*, *Raphanus sativus* and *Arabis alpina*) were obtained from their complete cpDNA sequences (Genbank ID: GQ861354.1, KJ716483.1 and NC023367.1) by using BLAST. *Eutrema salsugineum* and *A. alpine* were used as outgroups of the *ZIP* gene. The homologous sequences of the *ZIP* gene were identified from the whole-genome sequences of *E. salsugineum* and *A. alpina* separately by using BLAST. The tree results were finally displayed and edited using FigTree version 1.3.1 [62]. Chloroplast haplotypes and nuclear alleles networks for cpDNA and nDNA marker were estimated using median-joining networks with NETWORK version 4.6.1.1 [63].

### 4.5. Divergence Time Estimation

Divergence times between *T. arvense* and outgroup species were estimated by a study of molecular dating in Brassicaceae [30]. In this study, Beilstein used four fossil calibrations, including *Thlaspi primaevum* which is close to *T. arvense.* According to the dating result (figure S4 of [30]), 38.4 Ma (33.2–45.0 Ma) and 35.9 Ma (31.1–41.7 Ma) were used as the divergence time between *T. arvense* and outgroups (*B. napus*, *R. sativus* and *A. alpina* for cpDNA, and *E. salsugineum* and *A. alpine* for the *ZIP* gene), separately. For both cpDNA and nDNA datasets, a global molecular clock assumption was not rejected by molecular clock test in MEGA6 [64] by using BI tree as tree file. The haplotype divergent times were estimated by BASEML program of PAML version 4 [65] with global molecular clock (clock = 1). The dating results mentioned above (38.4 Ma for cpDNA and 35.9 Ma for *ZIP*) were positioned at the node between outgroups and *T. arvense* haplotypes in the BI tree as the guide tree of BASEML.

### 4.6. Population Genetic Diversity and Demography

Haplotype diversity (Hd) and nucleotide diversity (π) were calculated using DnaSP version 5.0 [59]. Two population differentiation parameters (G_ST_, N_ST_) were calculated using HAPLONST to infer if any phylogeographic structure exists [29]. If N_ST_ and G_ST_ differed significantly (N_ST_ > G_ST_), it indicates that the populations are phylogeographically structured. We also estimated haplotype frequency (hT), as well as both haplotype frequency and the genetic distance between haplotypes (vT). If hT > vT in areas with high allelic richness; the results indicated that the haplotypes may belong to the same genetic lineage. Otherwise, if hT ≤ vT, the region is suggested to be an admixture zone of subpopulations [35,36]. The above parameters in cpDNA and *ZIP* datasets were examined respectively in the whole population and in the populations of the eastern edge of the QTP. In order to test historical demographic dynamic state, we calculated the mismatch distributions and Tajima’s D by ARLEQUIN version 3.11 [66]. Mismatch distribution indicates the frequency distribution of the sites that differ between all unique pairs of DNA sequences. The shape of mismatch distribution can infer the history of the population [67]. Negative Tajima’s D means excess of low frequency variants, which indicates population growth or purifying selection. Positive value means excess of intermediate frequency variants, which indicates recent bottleneck, balancing selection or admixture between two highly divergent ancestral populations [37,38,39].

### 4.7. Palaeo-Distribution Modeling

Maximum entropy machine-learning algorithm was carried out by MaxEnt3.3.3k [27] to deduce the present and past potential geographic distribution of *T. arvense*. It began by obtaining 513 records from Chinese Virtual Herbarium (CVH, available online: http://www.cvh.org.cn/) and Global Biodiversity Information Facility (GBIF, available online: http://data.gbif.org/), as well as 19 records from this study. Because not all *T. arvense* records have GPS information in the CVH database, Google map was then used to search specific locations to identify coordinates. Bioclimatic variables of current condition (interpolations of observed data, representative of 1950–2000) were downloaded from WorldClim website (available online: http://www.worldclim.org/) [68] at 2.5 min resolution. Last Glacial Maximum (LGM; ~21,000 years BP) climate data simulated from the Community Climate System Model (CCSM) is also provided in the WorldClim website at 2.5 min resolution. We used default parameters to run MaxEnt. 75% of localities were used to train the model and then 25% for testing. Ten replicate runs were performed based on random subsampling of the calibration data set. The area under the curve (AUC) statistic was used to evaluate model performance [27].

## 5. Conclusions

We identified haplotypes (C4, C5, C7 and N4) specific to the QTP. Haplotype C4, C5 and C7 have separated from others during the QTP uplift. It is highly possible that the *T. arvense* population in China is a mixture of these highly diverged haplotypes. Populations from the eastern edge of the QTP contain the highest allelic richness, and at the same time, have the most stable climatic condition recognized by the result from MaxEnt modeling. However, the hT and vT test suggested that this region is likely to be an admixture zone of different genetic lineages rather than a refugia. Moreover, although the result from MaxEnt modeling suggested a northward range shift in *T. arvense* at lower altitudes, it failed to support recent demographic expansion as indicated by the results of the mismatch distribution. In short, our study reveals that the history of population dynamics is generally stable, but specific haplotypes have developed during the QTP uplift, and an admixture zone of genetic lineages is recognized in China.
